# The Divergent Effects of the Public’s Sense of Power on Donation Intention

**DOI:** 10.3390/bs13020118

**Published:** 2023-01-31

**Authors:** Yanpeng Yuan, Pingping Li, Fanghui Ju

**Affiliations:** 1 School of Public Affairs, Zhejiang University, Hangzhou 310058, China; 2 School of Marxism, NingboTech University, Ningbo 315100, China; 3 School of Business, NingboTech University, Ningbo 315100, China

**Keywords:** sense of power, donation intention, perceived ethical climate, regulatory focus theory

## Abstract

Studies of the relationship between individuals’ sense of power and donation intention have inconsistent findings. Classifying donor intention into two types, this study explored the mechanism through which a sense of power affects donation intention. Using a three-wave time-lagged survey of 1200 people, this study found that situational prevention focus mediates the positive effect of a sense of power on avoidance-based donation intention, and situational promotion focus mediates the positive effect of a sense of power on improvement-based donation intention. Furthermore, a strong perceived ethical climate strengthens the effects of a sense of power. These findings have practical implications for increasing charitable giving and improving the development of charitable programs.

## 1. Introduction

An important task for charitable organizations is to regulate widening global income gaps and to help the disadvantaged by encouraging charitable giving [1]. Charitable organizations have diverse types of programs to encourage public participation in charitable programs [2]. From a donor’s perspective, a donation is an act that makes an impact on society or improves society through the provision of goods or services. However, individuals’ sense of power and their judgment of the extent to which a single person can influence the environment may impact donation preferences [3,4]. Three related topics have not attracted the attention of researchers.

First, there have been no studies of the relationship between the sense of power and donation program preferences. Although some studies have found that high power is positively associated with proactive behavior [5,6], others have suggested that low power is positively associated with pro-social behavior [7,8]. Some studies have demonstrated that individuals’ coping strategies are strongly affected by their sense of power [9,10,11]. Based on this view, individuals with a high sense of power tend to adopt approach-based strategies to reach their goals [12], such as participating in uplifting projects and supporting environmental and social improvement projects (e.g., reforestation); individuals with a low sense of power tend to adopt avoidance-based strategies to achieve their goals [13], such as participating in avoidance-based projects that prevent the social environment from deteriorating (e.g., providing household utensils and clothing to the poor in disadvantaged communities). Unfortunately, there is a lack of research examining people’s giving behavior from the perspective of their sense of power.

Second, few studies have explored the mechanisms underlying the effects of a sense of power on donor program preferences. According to the approach-inhibition theory of power, a high sense of power stimulates higher levels of pro-social motivation, which in turn motivates individuals to engage in proactive behavior [14]. However, the social distance theory of power suggests that individuals with a higher sense of power are more indifferent toward others [15,16]. According to the self-regulatory focus model [12], individuals with a high sense of power are more sensitive to opportunities and tend to focus on how to optimize the current environment [17]. Thus, they tend to engage in uplifting projects that lead to the improvement of the social environment. Individuals with a low sense of power are more sensitive to threats and focus on how to avoid threats and the deterioration of the current situation [18]. Thus, they tend to participate in avoidance-based programs that prevent the social environment from deteriorating. However, the theoretical framework explaining the impact of high and low power differences on people’s giving behavior needs to be improved.

Third, because individuals have different levels of sense of power and different levels of interest in charitable projects, the boundary conditions of the effects of a sense of power on charitable projects are unclear. Thus, scholars have called for an examination of the contextual factors that shape the relationship between sense of power and donation intention [3,19]. In an environment with a strong ethical climate, individuals with a promotion focus see optimizing the environment and promoting positive social developments as an important way to fulfill their ethical responsibilities [20] and, thus, exhibit higher levels of participation in uplifting programs. In contrast, individuals with an inhibition focus see helping the disadvantaged as a key way to fulfill their ethical responsibilities [21] and, thus, exhibit higher levels of participation in avoidance-based giving programs. However, focusing on the field of social philanthropy, there is an urgent need to deepen research on the contextual mechanisms of the sense of power and people’s giving behavior from the perspective of ethical climate.

**Figure 1:**
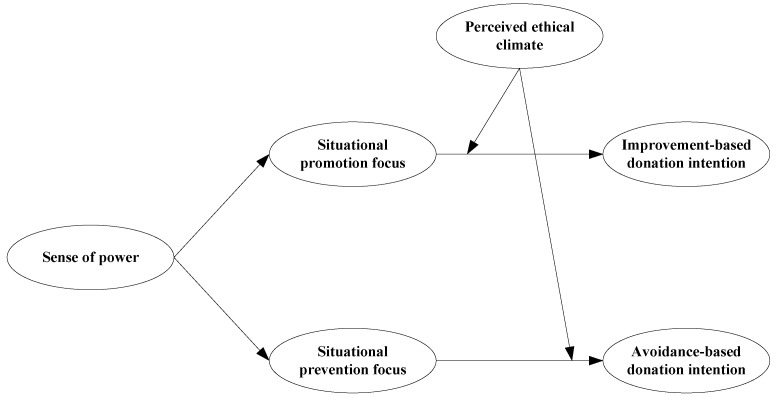
Hypothesized model.

Therefore, this study explored the mechanisms and contextual factors that shape the relationship between people’s sense of power and their participation in different types of giving programs (see Figure 1). It makes the following contributions. First, by separately considering the effects of a sense of power on preferences for approach-based giving programs and avoidance-based giving programs, this study deepens our understanding of the relationship between a sense of power and donation intention [22,23]. Second, this study used a situational regulatory focus model [12] to explore the mechanisms through which a sense of power affects approach-based and avoidance-based donation intentions, and this study enriches the theoretical research framework on the mechanisms of the role of sense of power on proactive behavior. Third, this study used perceived ethical climate as a contextual factor in a moderated mediation model, expanding our understanding of the boundary conditions of the relationship between sense of power and individual behavior [24] and providing insights that will help charitable organizations and online philanthropic crowdfunding platforms to improve their project presentation strategies.

## 2. Theory and Hypothesis

### 2.1. Regulatory Focus Theory

The regulatory focus model suggests [12] that people’s motivational orientations (e.g., whether they are interested or disgusted) are closely related to their surroundings. The two motivational orientations are promotion focus and prevention focus [25]. Individuals with a promotion focus are sensitive to positive information and tend to adopt approach strategies that allow them to pursue their “ideal” self-realization [26]. In contrast, individuals with a prevention focus are more sensitive to threats and losses, and they pursue fulfilment of their own duties, obligations, and responsibilities. They tend to adopt avoidance strategies [27]. Regulatory focus can be not only a personal trait but also a motivational state that is regulated by the environment [28].

Individuals with a high sense of power are more sensitive to opportunities in the environment and are less affected by environmental constraints than individuals with a low sense of power, who are more sensitive to threats in the environment and are more influenced and constrained by their environments [9,29,30]. According to the regulatory focus model, individuals with a high sense of power focus on opportunities and access in their social environments [31,32] and are willing to change the status quo to optimize social conditions and improve the standard of living of society as a whole [9]. As a result, when choosing a donation program, they have a strong preference for enhancement projects, such as improving specific social groups’ access to art (e.g., optimizing elementary school students’ reading or promoting Chinese traditional culture) or improving social and natural environments (e.g., air quality or urban greening projects). In contrast, individuals with a low sense of power are more focused on threats and losses and tend to think about how to avoid losses and negative outcomes [33]. They are concerned with environmental degradation and low standards of living among the general public [9]. As a result, when choosing a donation program, they have a strong preference for avoidance-based donation programs, such as relief for the disadvantaged (e.g., assistance programs for widows and orphans or for mentally challenged children), supporting living conditions in disadvantaged areas and improving the quality of life of social groups in economically disadvantaged areas (e.g., drinking water projects in drought-stricken areas, housing and transportation projects in disadvantaged areas). Extending regulatory focus theory, this study examined the mechanisms and situational conditions that affect the relationship between power levels and charitable program preferences through a situational regulatory focus.

### 2.2. Sense of Power and Donation Intention

Donors with a high sense of power prefer facilitative giving (i.e., donation to enhancement projects). A high sense of power motivates individuals to focus on opportunity and access and to use facilitative strategies to achieve target outcomes. Conversely, donors with a low sense of power are more concerned with threats and losses and adopt avoidance strategies to achieve target outcomes [29,34]. Therefore, individuals with a high sense of power prefer to make donations to projects that enhance the environment and optimize environmental conditions, whereas individuals with a low sense of power donate to charitable projects that prevent the deterioration of social conditions. Some studies have shown that individuals with a high sense of power tend to pay more attention to positive information, whereas individuals with a low sense of power pay more attention to negative information [31,35,36]. Other studies have shown that individuals with a high sense of power tend to adopt positive strategies to optimize environmental conditions, such as proactively suggesting actions that will optimize an environment, whereas individuals with a low sense of power tend to adopt defensive strategies and make suggestions for avoiding making an environment worse [37]. Therefore, we made the following hypotheses.

**H1a:**  *Sense of power is positively associated with improvement-based donation intention.*

**H1b:**  *Sense of power is negatively correlated with avoidance-based donation intention*.

### 2.3. Mediating Role of Contextual Regulatory Focus

A high sense of power can promote a situational promotion focus. Individuals with a high sense of power are sensitive to opportunities. They are more inclined to pursue their ideal selves [38] and to focus on ways to optimize the status quo or change the status quo so that they can achieve self-performance goals. Their focus on the positive results of philanthropic projects and on how donations can optimize their life goals stimulates a situational promotion focus. In contrast, the focus of individuals with a low sense of power on how their donations may solve social problems stimulates a contextual prevention focus. Organizational research has also shown that individuals with a high sense of power focus on making organizational working conditions better, whereas individuals with a low sense of power focus on reducing inefficiencies [37].

Situational promotion focus is positively related to a preference for giving to projects that enhance social and natural environments (enhancement preference), while a situational prevention focus is positively related to a preference for giving to projects that reduce social problems (avoidance preference). Promotion focus makes individuals sensitive to positive messages and likely to adopt facilitative strategies that optimize environmental conditions [39]. Individuals whose promotion focus is stimulated tend to adopt facilitative strategies to achieve their goals, i.e., they tend to prefer to donate to enhancement-oriented projects that optimize current social and environmental conditions such as improving the environment, art education, and loans for improving people’s health. Research has shown that stimulating individuals’ situational promotion focus then leads to promoting optimization and the improvement of environmental conditions [40]. Therefore, we hypothesized that individuals with a high sense of power focus on promoting positive change in the donation process and thus donate to uplifting donation programs. Thus, we made the following hypothesis.

**H2a:**  *Situational promotion focus mediates the indirect effect of a sense of power on improvement-based donation intention.*

In contrast, prevention messages in donation campaigns may trigger individuals’ situational prevention focus; such individuals tend to use inhibitory strategies to achieve their goals, and they prefer to donate to avoidance-based programs, such as assistance programs for widows and orphans or for mentally challenged children, drinking water projects in drought-stricken areas, or housing and transportation projects in disadvantaged areas. Research has shown that triggering individuals’ situational prevention focus then directs attention to programs that protect the environment or address potential losses [41]. Therefore, we hypothesized that a low sense of power stimulates individuals’ situational prevention focus, which in turn promotes a preference for avoidance-based donation programs. Therefore, we made the following hypothesis.

**H2b:**  *Situational prevention focus mediates the indirect effect of a sense of power on avoidance-based donation intention.*

### 2.4. Mediating Effects of the Perceived Ethical Climate

The perceived ethical climate reinforces the effect of situational promotion focus on preferences for improvement-based programs. The perceived ethical climate is an individual’s perception of the norms and social practices shared by group members [42]. It regulates individual behavior by communicating to group members “what behaviors are widely accepted and promoted in the group” and “what behaviors should be avoided and rejected by the group”. Research has shown that ethical climate not only moderates the effect of individual fears on their behavioral preference choices [19], but it also attenuates the positive effects of leadership self-interest on employees’ tendency to retaliate [43]. A positive perceived ethical climate sends a message to people confronted with a donation campaign that “it is important to care about the interests of other members of their group”, and individuals can adjust their strategies to achieve their goals under this condition. Research has indicated that an ethical climate can guide individuals’ strategic choices in giving [44].

Individuals with a high sense of power are more sensitive to opportunities and acquisitions and focus on optimizing environmental conditions to achieve their ideal selves [45]. Thus, they have a situational promotion focus. When placed in a strong ethical climate and confronted with diverse giving options, individuals with a situational promotion focus realize that caring for others’ interests is an important way to optimize social environment and achieve their desired goals. Thus, they prefer donating to uplifting programs. Conversely, when placed in a weak ethical climate and confronted with diverse giving options, individuals with a situational promotion focus recognize that the interests of others are not directly related to the achievement of self-desired goals and the optimization of environmental conditions. Thus, a weak ethical environment reduces their preference for giving. Therefore, we made the following hypothesis.

**H3a:**  *The perceived ethical climate moderates the indirect effect of sense of power on improvement-based donation intention through situational promotion focus. Specifically, the perceived ethical climate is positively related to the indirect effect of sense of power on improvement-based donate intention through situational promotion focus.*

Individuals with a low sense of power are more sensitive to threats and losses and focus on avoiding damage to fulfill their sense of self-responsibility[9]. In a strong perceived ethical climate, this situational prevention focus inclines individuals to recognize that helping the socially disadvantaged is a group norm. Thus, they see giving to a charity that helps people in difficult situations as a way to fulfill their personal responsibility, and this leads to a preference for avoidance-based giving programs. Conversely, when in a weak perceived ethical climate, individuals with a situational prevention focus recognize that fulfilling their social responsibility is not directly related to group norms. Thus, they are less likely to donate to programs. Research has also shown that an ethical climate weakens the positive effects of self-interested leadership behaviors on employees’ tendency to retaliate [43].

**H3b:**  *The perceived ethical climate moderates the indirect effect of a sense of power on avoidance-based donate intention through contextual preference focus. Specifically, the perceived ethical climate is positively related to the indirect effect of a sense of power on avoidance-based donation intention through situational prevention focus.*

## 3. Method

### 3.1. Sample

The sample used in this study consisted of 1125 randomly selected people from a coastal province in eastern mainland China. The province is a developed region in China and a model region for charitable giving in China. The charitable sector in this region has more experience in managing charitable activities, with the result that it achieves a high degree of sample fit to the research question. The specific sampling procedure was as follows. First, the participants were informed that the purpose of this study was to study social giving, that the findings would be used for academic purposes only and not for commercial interests, and that their personal information would not be shared. In the end, 1192 individuals agreed to take part in our questionnaire study. To avoid common method bias, a time-lagged design with three time points was used. Specifically, at the first time point, we asked the participants to report their sense of power, regulatory focus, and demographic variables (including age, gender, and education level). After 4 weeks, at the second time point, the participants were asked to report their situational regulatory focus and the perceived ethical climate. After another 4 weeks, at the third time point, the participants were asked to report their improvement-based donation intention and avoidance-based donation intention. After three rounds of measurement, 1125 participants completed the questionnaire (94.38% completed). The majority of the participants were male (66.3%), the average age was 38.4 years (*SD* = 12.55), and 25.4% of the participants had college or higher education.

### 3.2. Measures

All of the variables were translated and back-translated to ensure their validity [46]. The perceived ethical climate was measured using a 7-point Likert scale (1 = *strongly disagree* to 7 = *strongly agree*), and all of the other items were assessed on 5-point Likert scales (1 = *strongly disagree* to 5 = *strongly agree*).

***Sense of power*.** The 8-item scale developed by Anderson, John, and Keltner [29] was used to measure individuals’ sense of power. Sample items are “In daily life, if I want to, I get to make the decisions” and “In daily life, even when I try, I am not able to get my way” (revised coded) (α = 0.90).

***Situational regulatory focus.*** We used 10 items from a survey developed by Lockwood, Jordan, and Kunda [47] and, following other studies, adapted them to measure situational regulatory focus [48,49]. Five items measured situational prevention focus (α = 0.89), and five measured situational promotion focus (α = 0.90). A sample item for situational prevention focus is “As a member of the community, I am worried that I will not meet my work obligations and responsibilities in my daily life,” and a sample item for situational promotion focus is “As a member of the community, I often think about how I will improve society”.

***Improvement-based donation intention*.** We measured improvement-based donation intention using a 4-item scale for willingness to donate [50,51]. The scale’s introduction is as follows: *Improvement-based donation projects aim to improve the current state of society, for example, by optimizing air quality, enhancing the reading habits of primary school students, or promoting traditional culture*. A sample item is “I wish to donate money to this type of project as soon as possible” (α = 0.88).

***Avoidance-based donation intention*.** We used four items adopted from the willingness to donate scale [50,51] to measure avoidance-based donation intention. The introduction is as follows: *Avoidance-based donation projects aim to avoid the deterioration of the social status quo, for example by assisting widows and orphans, reinstating out-of-school children, or providing access to drinking water for remote communities*. A sample item is “I wish to donate money to this type of project as soon as possible” (α = 0.89).

***Perceived ethical climate*.** Five items developed by Barnett and Vaicys [52] were used to measure the perceived ethical climate. A sample item is “In our local area, people have a sense of responsibility for the people around them” (α = 0.92).

***Control variables.*** Following Liu, Yuan, Lu, and Ju [22], we controlled for the following demographic variables: age, gender, and education.

## 4. Results

A confirmatory factor analysis found a good fit between the observed data and our hypothesized model (χ^2^ = 179.31, *df* = 120, comparative fit index [CFI] = 0.99, Tucker-Lewis index (TLI) = 0.99, root mean square error of approximation (RMSEA) = 0.02, standardized root mean square residual (SRMR) = 0.02), indicating satisfactory discriminant validity. The means, standard deviations, and correlations between the variables are displayed in Table 1. Sense of power was significantly positively related to situational promotion focus (β = 0.41, *p* < 0.01) and improvement-based donation intention (β = 0.32, *p* < 0.01), and it was negatively associated with situational prevention focus (β = −0.35, *p* < 0.01) and avoidance-based donation intention (β = −0.09, *p* < 0.01). Furthermore, situational promotion focus was positively related to improvement-based donation intention (β = 0.44, *p* < 0.01), and situational prevention focus was positively related to avoidance-based donation intention (β = 0.32, *p* < 0.01). These results provided the basis for testing our hypotheses.

After controlling for age, gender, and education, the results (See Table 2) revealed that sense of power was positively related to improvement-based intention (β = 0.34, *p* < 0.01) and negatively related to avoidance-based donation intention (β = −0.10, *p* < 0.01), supporting Hypotheses 1a and 1b. An analysis of 20,000 bootstrap samples found that the indirect effect for sense of power → situational promotion focus → improvement-based donation intention was 0.16, and the 95% confidence intervals (CI) did not contain zero (CI = [0.124, 0.194]). The indirect effect for sense of power → situational prevention focus → avoidance-based donation intention was −0.12, and the 95% CI did not contain zero (CI = [−0.145, −0.093]). Therefore, Hypothesis 2a and 2b were also supported.

As shown in Table 3, the indirect effect of sense of power on improvement-based donation intention through situational promotion focus was significant for individuals in both the high perceived ethical climate group (γ = 0.22, CI = [0.175, 0.266]) and the low perceived ethical climate group (γ = 0.16, CI [0.120, 0.200]), but there was a significant difference in the effect (γ = 0.06, CI = [0.017, 0.104]), supporting Hypothesis 3a. The indirect effect of sense of power on avoidance-based donation intention via situational prevention focus was significant for individuals in the high perceived ethical climate group (γ = −0.18, CI = [−0.220, −0.148]), but this indirect effect was not significant for individuals in the low perceived ethical climate group (γ = −0.05, CI = [−0.073, −0.020]). The difference between the high perceived ethical climate and low perceived ethical climate groups was significant (γ = −0.14, CI = [−0.180, −0.096]), supporting Hypothesis 3b.

*P*M1X refers to the path from sense of power to situational promotion focus; *P*Y2M2 refers to the path from sense of power to situational prevention focus. *P*Y1M1 refers to the path from situational promotion focus to improvement-based donation intention; *P*Y2M2 refers to the path from situational prevention focus to avoidance-based donation intention. Diff refers to the moderated mediation effect difference between perceived ethical climate with high level and low level.

CI refers to confidence interval. Confidence intervals were calculated using bootstrap sampling.

## 5. Discussion

Using a three-wave time-lagged design, this study found that situational promotion focus mediates the positive indirect effect of sense of power on improvement-based donation intention, and situational prevention focus mediates the negative indirect effect of sense of power on avoidance-based donation intention. Furthermore, perceived ethical climate enhances the effect of situational promotion focus on improvement-based donation intention and the indirect effect of a sense of power on improvement-based donation intention via situational promotion focus. Perceived ethical climate enhances the effect of situational prevention focus on avoidance-based donation intention and the indirect effect of a sense of power on avoidance-based donation intention through situational prevention focus. These findings make theoretical contributions to the literature and have some practical implications.

### 5.1. Theoretical Contributions

First, focusing on the context of charitable giving in society, this study separately examined the impact of sense of power on improvement-based donation intention and avoidance-based donation intention and enriched research contexts of sense of power on individual behavior. Some studies have found that a sense of power is positively related to proactive behavior [9,53,54], but others have found that individuals with a low sense of power exhibit generous behaviors [7]. Meanwhile, some studies have found inconsistencies in the relationship between a sense of power and proactive behavior [33]. For example, sense of power has been found to stimulate individuals’ perceptions of responsibility when there is a high level of ethical identity, which in turn positively influences giving behavior [3,22]. This study explored the different effects of a sense of power on improvement-based and avoidance-based donation intentions, and found that the different effects on the two types of donation intentions can explain the inconsistent findings on the relationship between a sense of power and donation intention in the literature. Thus, by distinguishing between two different types of donor behavior, this study explains some reasons that account for inconsistent findings on the relationship between sense of power and proactive behavior and thus deepens our understanding of the relationship between a sense of power and donation behavior.

Second, this study used self-regulatory focus theory to test the mechanism through which a sense of power affects donation intention and enriched research on the mechanisms through which a sense of power influences individual behavior. According to the approach-inhibition theory of power, a sense of power motivates individuals to engage in proactive behavior [9]; however, according to the distance theory of power, a high sense of power increases social distance, which in turn motivates self-interested behavior or less altruistic behavior [8,55]. Using the regulatory focus model, this study found that individuals with a high sense of power focus on optimizing the environment and tend to participate in enhancement philanthropy projects that improve the social environment, whereas individuals with a low sense of power focus on how to avoid threats and tend to participate in avoidance philanthropy projects that prevent the deterioration of the social environment. Thus, this study not only explains why a sense of power has divergent effects on improvement-based and avoidance-based donation intentions, but it also enriches our understanding of the mechanism through which sense of power affects proactive behavior.

Third, by introducing ethical climate as a mediating factor, this study revealed the boundary conditions of the relationship between sense of power and donation intention. Other studies have shown that power motivation can weaken the positive influence of a sense of power on risky behavior [33], while ethical identity can strengthen the positive impact of a sense of power on proactive behavior [8]. It has also been found that an ethical climate can guide the choice of behavioral strategies of individuals who are fearful of emotions [19]. Focusing on people’s giving behavior in the social charity field, this study showed that an ethical climate can amplify the indirect effects of individuals’ sense of power on improvement-based donation intention through situational promotion focus, and it can foster the indirect effects of individuals’ sense of power on avoidance-based donation intention. Thus, compared to previous studies [22,56], this research revealed the boundary conditions for the effects of a sense of power on donation intention and enhanced our understanding of the explanatory power of ethical climate, and it not only enhanced the explanatory power of ethical climate [57], but it also expanded the study of the boundary conditions of the relationship between the sense of power and individual behavior [29].

### 5.2. Practical Implications

The findings of this study have several implications for management practices in the social giving sector. First, this research indicates that a sense of power has different effects on improvement-based and avoidance-based donation intentions. Therefore, charity management can take some measures to stimulate individuals’ donation behavior by influencing their sense of power. Specifically, in the promotion of charitable activities, when fundraising for uplifting projects, management can stimulate individuals’ sense of power by emphasizing the importance of optimizing environmental conditions and their significance to society. That is, increasing individuals’ sense of control over the environment will enhance their improvement-based donation intention [9,15,58]. Charitable organizations can target groups with better-off classes when fundraising for improvement-based programs. Previous research believed that the group with better-off classes has a higher sense of power [59,60]. At the same time, during the avoidance-based donation activities, the charity publicity department can appropriately reduce the people’s sense of power, such as making them realize that they are only a member of the general public and belong to the same group as recipients of donations [29], thus increasing the involvement of the public in social charity activities.

Second, this study found that a sense of power stimulates individuals’ situational promotion focus, which in turn positively influences their improvement-based donation intention. In contrast, by stimulating individuals’ situational prevention focus, a sense of power negatively influences avoidance-based donation intention. Thus, when fundraising for uplifting charitable projects, philanthropic organizations can introduce management measures that enhance people’s contextual promotion focus; that is, they can educate people about the positive effects of donations on society. Conveying positive messages can promote individuals’ situational promotion focus [48]. When fundraising for avoidance-based projects, stewardship measures can be introduced to enhance the public’s situational prevention focus; that is, these organizations can inform people that not donating may lead to the deterioration of the social environment. Conveying negative information to individuals can stimulate their situational prevention focus [49].

Third, this study found that an ethical climate can reinforce the positive effect of a sense of power on improvement-based donation intention through situational promotion focus and can foster the indirect effect of a sense of power on avoidance-based donation intention through situational prevention focus. Therefore, the philanthropic sector can publicly strengthen the ethical climate to amplify the effect of a sense of power on donation intention. For example, community management can invite people to watch films related to traditional Chinese culture to promote awareness of the social importance of caring for their interests and to create a positive ethical atmosphere among the population. Meanwhile, charity management can also use the internet to communicate the importance of concern for the interests of others and to promote a positive ethical climate among society at large. Previous research argued that organizations could directly promote the ethical climate and charity culture by communicating to the public that “caring for the interests of others is strongly advocated by social norms” [19] and help cultivate a strong ethical climate.

## 6. Limitations and Future Directions

This study has several limitations. First, although it used a three-wave time-lagged design, all of the variables are self-reported, which may result in social desirability bias [61]. Thus, future studies may consider using a combination of self-reported and other-rated measures to avoid common method and social desirability biases [62,63]. Second, this study provides evidence of the mediating role of situational regulatory focus, but there may be other alternative mechanisms through which a sense of power influences improvement-based and avoidance-based donation intentions. For example, it has been suggested that sense of power is positively related to positive emotions and negatively related to negative emotions [64] and that these emotions have differential effects on proactive behavior [65,66]. Therefore, future research could examine whether power affects improvement-based and avoidance-based donation intentions through positive or negative emotions.

## 7. Conclusions

This study shows that a high sense of power stimulates a situational promotion focus, which increases improvement-based donation intention, and that a low sense of power stimulates a situational prevention focus, which increases avoidance-based donation intention. Furthermore, the effect of sense of power on donation intention through situational regulatory focus is stronger when there is a strong (versus weak) perceived ethical climate.

## Figures and Tables

**Table 1:** Means, standard deviations, and correlations.

Variables	M	SD	1	2	3	4	5	6	7	8	9
1. Age	38.40	12.55									
2. Gender	0.67	0.47	0.09 **								
3. Education	3.20	0.53	−0.18 **	−0.05							
4. Sense of power	3.30	0.82	−0.01	0.03	−0.01						
5. Situational promotion focus	3.49	0.90	0.02	0.00	−0.02	0.41 **					
6. Situational prevention focus	3.45	0.91	−0.02	0.04	−0.03	−0.35 **	−0.12 **				
7. Improvement-based donation intention	3.60	0.87	−0.02	0.01	0.01	0.32 **	0.44 **	−0.10 **			
8. Avoidance-based donation intention	3.90	0.87	−0.03	0.02	−0.03	−0.09 **	0.08 **	0.32 **	0.02		
9. Perceived ethical climate	4.37	1.10	0.01	0.03	−0.03	−0.01	0.03	0.08 *	0.12 **	0.17 **	

Note: *N* = 1125. * *p* < 0.05; ** *p* < 0.01.

**Table 2:** Direct and indirect effects of sense of power on improvement-based donation intention and avoidance-based donation intention.

	Situational Promotion Focus	Situational Prevention Focus	Improvement-Based Donation Intention	Avoidance-Based Donation Intention
Variable name	*B (SE)*	*B (SE)*		
Controls				
Age	0.00 (0.00)	−0.00 (0.00)	−0.00 (0.00)	−0.00 (0.00)
Gender	0.02 (0.05)	0.05 (0.06)	0.04 (0.05)	0.04 (0.05)
Education	−0.03 (0.05)	−0.07 (0.05)	0.01 (0.05)	−0.05 (0.05)
Direct effects				
Sense of power	0.45 ** (0.03)	−0.39 ** (0.03)	0.34 ** (0.03)	−0.10 ** (0.03)
Situational promotion focus			0.35 ** (0.06)	
Situational prevention focus				0.31 ** (0.03)
Mediating effects			Point estimate [95% CI]; 20,000 bootstrapping sampling	
Sense of power → SPOF → IDI			0.16 [0.124, 0.194]	
Sense of power → SPEF → ADI				−0.12 [−0.145, −0.093]

Note: *N* = 1125. ** *p* < 0.01. SPOF refers to situational promotion focus, SPEF refers to situational prevention focus, IDI refers to improvement-based donation intention, ADI refers to avoidance-based donation intention.

**Table 3:** Moderated mediation effect of perceived ethical climate.

		Stage			Effect
Outcome	Moderator: Perceived ethical climate	First (*P*M1X)	Second (*P*Y1M1)	*Indirect* (*P*M1X * *P*Y1M1)	95% CI of Indirect effect, 20,000 bootstrap sampling
Improvement-based donation intention	Low (−1 SD)	0.45 ** (0.03)	0.35 ** (0.03)	0.16 ** (0.02)	[0.120, 0.200]
	High (+1 SD)		0.49 ** (0.04)	0.22 ** (0.02)	[0.175, 0.266]
	Diff	0.13 ** (0.05)		0.06 ** (0.02)	[0.017, 0.104]
	Moderator: Perceived ethical climate	First (*P*M2X)	Second (*P*Y2M2)	*Indirect* (*P*M2X * *P*Y2M2)	95% CI of Indirect effect, 20,000 bootstrap sampling
Avoidance-based donation intention	Low (−1 SD)	−0.39 ^**^ (0.03)	0.12 ** (0.04)	−0.05 (0.01)	[−0.073, −0.020]
	High (+1 SD)		0.48 ** (0.03)	−0.18 (0.02)	[−0.220, −0.148]
	Diff	0.36 ^**^ (0.05)		−0.14 (0.02)	[−0.180, −0.096]

Note: *n* = 1125. ** *p* < 0.01.

## Data Availability

The data that support the findings of this study are available on request from the corresponding author.
